# Detection of Soluble ED-A^+^ Fibronectin and Evaluation as Novel Serum Biomarker for Cardiac Tissue Remodeling

**DOI:** 10.1155/2016/3695454

**Published:** 2016-08-18

**Authors:** Barbara Ziffels, Johanna Ospel, Katja Grün, Dario Neri, Alexander Pfeil, Michael Fritzenwanger, Hans R. Figulla, Christian Jung, Alexander Berndt, Marcus Franz

**Affiliations:** ^1^Jena University Hospital, Department of Internal Medicine I, 07747 Jena, Germany; ^2^Swiss Federal Institute of Technology, Department of Chemistry and Applied Biosciences, 8093 Zurich, Switzerland; ^3^Jena University Hospital, Department of Internal Medicine III, 07747 Jena, Germany; ^4^Medical Faculty, Division of Cardiology, Pulmonology, and Vascular Medicine, University of Duesseldorf, 40225 Duesseldorf, Germany; ^5^Jena University Hospital, Institute of Pathology, 07743 Jena, Germany

## Abstract

*Background and Aims*. Fibronectin containing the extra domain A (ED-A^+^ Fn) was proven to serve as a valuable biomarker for cardiac remodeling. The study was aimed at establishing an ELISA to determine ED-A^+^ Fn in serum of heart failure patients.* Methods*. ED-A^+^ Fn was quantified in serum samples from 114 heart failure patients due to ischemic (ICM, *n* = 44) and dilated (DCM, *n* = 39) cardiomyopathy as well as hypertensive heart disease (HHD, *n* = 31) compared to healthy controls (*n* = 12).* Results*. In comparison to healthy volunteers, heart failure patients showed significantly increased levels of ED-A^+^ Fn (*p* < 0.001). In particular in ICM patients there were significant associations between ED-A^+^ Fn serum levels and clinical parameters, for example, increased levels with rising NYHA class (*p* = 0.013), a negative correlation with left ventricular ejection fraction (*p* = 0.026, *r*: −0.353), a positive correlation with left atrial diameter (*p* = 0.008, *r*: 0.431), and a strong positive correlation with systolic pulmonary artery pressure (*p* = 0.002, *r*: 0.485). In multivariate analysis, ED-A^+^ Fn was identified as an independent predictor of an ischemic heart failure etiology.* Conclusions*. The current study could clearly show that ED-A^+^ Fn is a promising biomarker in cardiovascular diseases, especially in heart failure patients due to an ICM. We presented a valid ELISA method, which could be applied for further studies investigating the value of ED-A^+^ Fn.

## 1. Introduction

Diseases of the cardiovascular system are the most frequent cause of death worldwide and essentially contribute to morbidity especially in elderly patients [1]. Thus, there is a high clinical interest to develop novel and sufficient strategies to prevent the disease, to diagnose it at an early stage, and to establish sufficient therapies. Specific cardiomyopathies, in particular ischemic cardiomyopathy, hypertensive or valvular heart disease, and dilated cardiomyopathy are accompanied by structural and functional alterations of the heart muscle. This so-called myocardial remodeling initially enables compensatory mechanisms to maintain cardiac function despite the persisting pathologic stimuli. At later stages, the remodeling process proceeds to left ventricular dysfunction with the clinical consequence of the heart failure syndrome. Especially in case of hypertensive heart disease (HHD) but also in ischemic cardiomyopathy (ICM), heart failure can present in two different forms: heart failure with reduced ejection fraction (HFrEF) or heart failure with preserved ejection fraction (HFpEF). Usually, HFpEF is present in the stage of compensation, that is, left ventricular hypertrophy in case of HHD, and is represented by a diastolic left ventricular dysfunction while systolic function is still normal or only mildly reduced. Nevertheless, even at this stage severe heart failure symptoms can occur [2, 3]. Myocardial remodeling is a complex and heterogeneous process involving cardiac myocyte alterations, activation of fibroblasts, and transdifferentiation to myofibroblasts (MyoFb) as well as a dynamic functional and structural reorganization of the cardiac extracellular matrix (cECM) [2, 4–6]. Whereas several novel treatment strategies focus on cardiac myocytes or inflammation, only very limited attention is paid to the MyoFb and fibrosis development as well as cECM reorganization [3]. Reorganization of cECM is accompanied by a reoccurrence of fetal variants of large cell adhesion glycoproteins like fibronectin or tenascin-C. These molecular variants are overexpressed in critical stages of heart development, are virtually absent in healthy adult organs, and show an extensive reexpression in association with myocardial remodeling [7–12]. Thus, they qualify as both excellent biomarkers for diagnosis or disease progression monitoring and therapeutic targets by means of antibody based delivery of bioactive payloads or drugs directly to the site of disease. Moreover, considering their disease promoting capacity, also functional blocking of the molecules seems to be promising novel strategy to address pathologic tissue remodeling [13–15]. Most studies evaluating the applicability of fetal variants of cell adhesion molecules in cardiovascular diseases have investigated serum levels of different tenascin-C splicing variants [16–24]. When investigating the reoccurrence of fetal splicing variants of fibronectin in cardiovascular diseases, one could learn that especially the extra domain A of fibronectin (ED-A^+^ Fn) can be frequently detected in diseased cardiac tissue from patients with ischemic cardiomyopathy, valvular heart disease, and dilated cardiomyopathy and also in a rat model of chronic cardiac rejection by our group [25–30]. Against that background and due to its stable tissue deposition, the molecule could be shown to be a robust target for antibody based delivery of diagnostic agents or bioactive payloads using the human ED-A domain specific antibody F8 as a vehicle [31, 32]. In addition, it could recently be shown that fibronectin in general may serve as a serum biomarker in several diseases like viral hepatitis, pulmonary tuberculosis, Duchenne muscular dystrophy, and melanoma [33, 34]. There are only very limited data available on the role of ED-A^+^ Fn as a diagnostic biomarker to detect cardiovascular diseases or to monitor its progression/therapy response (reverse cardiac remodeling). To our best knowledge, there is only one study available, which could demonstrate that the tissue deposition of ED-A^+^ Fn in left ventricular biopsies of patients presenting with idiopathic dilated cardiomyopathy is of prognostic relevance [27]. There are no studies on the possible impact of the molecule as a serum biomarker. This might, at least in part, be due to the fact that there is no commercially available ELISA to determine ED-A^+^ Fn in body fluids, in particular in patients' serum.

## 2. Aims of the Study

Motivated by these hitherto open questions, the aim of our current study was first to establish a sensitive and specific ELISA protocol to reliably determine ED-A^+^ Fn serum levels in human blood samples and second to present a proof of principle analysis of the amount of ED-A^+^ Fn that is liberated into the circulation in heart failure patients with reduced left ventricular ejection fraction (HFrEF) or preserved left ventricular ejection fraction (HFpEF) as a consequence of different underlying pathologies, namely, ischemic cardiomyopathy, hypertensive heart disease, and dilated cardiomyopathy.

## 3. Methods

### 3.1. ED-A^+^ Fn ELISA Protocol, Generation of a Standard Curve, and Specificity Evaluation

The establishment of an appropriate ELISA protocol to specifically detect the ED-A domain of fibronectin in serum based on the following methodological consideration: the N-terminal end of the fibronectin molecule contains a gelatin binding site, which has been described to serve as a valuable structure to capture the whole molecule by a gelatin coated plate for effective quantification [35]. Following the principle of an indirect ELISA, in the second step, the ED-A domain could be detected using a domain specific primary antibody and a biotinylated secondary antibody.

The optimization of the ELISA procedure for quantification of soluble ED-A^+^ Fn concentration in serum samples of heart failure patients resulted in the following final protocol: Coating of the ELISA plates (96-well plate, high binding, F-Bottom ELISA Microplate, Greiner Bio-One GmbH, Frickenhausen, Germany) was performed with 300 *μ*L 0.5% gelatin in PBS/well overnight at 4°C. The following steps were performed at 20°C and all respective samples were diluted in PBS. Between the single steps of incubation, three washing procedures with 300 *μ*L PBS/0.1% Tween each were performed. In a next step, 50 *μ*L of the protein/serum samples (diluted in PBS, range 1 : 2 to 1 : 8) were added and allowed to incubate for one hour. For the detection of bound ED-A domain containing Fn, an incubation with 100 *μ*L IST-9/well (1.0 *μ*g/mL, Sirius Biotech S.r.l., Genoa, Italy) followed for one hour. Then, 100 *μ*L of a highly absorbed biotinylated donkey-*α*-mouse antiserum (715-066-151, diluted 1 : 10000, Dianova GmbH, Hamburg, Germany) was added again for one hour. Finally, 100 *μ*L of horseradish-peroxidase- (HRP-) labeled streptavidin (1.5 *μ*g/mL, Dianova GmbH, Hamburg, Germany) was added and incubated for 40 min. Detection was performed using H_2_O_2_ and O-phenylenediamine dihydrochloride (OPD) as chromogenic substrate. For that purpose, OPD-tablets (2 mg, Dako, Hamburg, Germany) were dissolved and used following the manufacturer's instructions. The reaction was stopped by use of 100 *μ*L 0.5 M H_2_SO_4_. Absorbance was read at a wavelength of 490 nm (Tecan Sunrise Reader, Tecan Group, Männedorf, Switzerland).

To generate a standard curve with a linear range, pure human ED-A^+^ Fn (S-P004; purified from spent melanoma cell line supernatant by 2-step affinity chromatography using gelatin and the antibody IST-9, Sirius Biotech, Genoa, Italy) as well as cellular fibronectin (F2518, isolated from human foreskin fibroblasts with an unknown procedure of purification, Sigma-Aldrich, Missouri, USA) were available and applied in a range from 0.078 *μ*g/mL to 10 *μ*g/mL to a 0.5% gelatin coated ELISA plate. After this, detection and visualization (primary antibody: IST-9; secondary antibody: biotinylated donkey-*α*-mouse antibody) were performed as stated previously in this section.

For exclusion of cross-reactions between any of the components enclosed in the newly established ELISA, the protocol was tested with different variants: a “complete” protocol was compared with 4 modified experimental setups with one missing component each. As ED-A^+^ Fn containing positive control, 50 *μ*L of serum-free supernatant of TGF-*β*1 stimulated hTERT-BJ1 fibroblasts was used as described above. The supernatant was prepared as described earlier by our group [36]. ED-A^+^ Fn synthesis, extracellular deposition, and release into the medium were evidenced by immunohistochemistry and western blotting (data not shown). pFn, which is synthesized by hepatocytes, does not contain the extra domain A and is directly released into the blood stream [37] and should not be detected by the ELISA. To prove detection specificity of the established assay, Fn isolated from human plasma (F2006, Sigma-Aldrich, Missouri, USA) was used in parallel to ED-A^+^ Fn from melanoma cell and fibroblast supernatant in a concentration range from 0.078 *μ*g/mL to 10 *μ*g/mL in the same setup as used for standard curve establishment as described above.

For exclusion of unspecific binding of the secondary antibody to endogenous immunoglobulins in the serum samples, the ELISA as described above was performed with and without the detection antibody IST-9 using a human serum sample in a dilution range from 1 : 2 to 1 : 8. Signal detection was performed as described before. Measurements were performed in duplicate. Data in the graphs are given as mean ± standard deviation if appropriate/available.

### 3.2. Quantification of ED-A^+^ Fn in Serum of Heart Failure Patients

For determination of circulating ED-A^+^ Fn, serum of 114 heart failure patients of different etiology was available. The patients were recruited from the cardiology outpatient department of Clinic of Internal Medicine I of Jena University Hospital. Moreover, 12 healthy volunteers served as control group. All patients and all healthy volunteers provided written informed consent to participate in the study, which has been approved by the local Ethics Committee of the Jena University Hospital and complies with the Declaration of Helsinki. Patients were eligible for inclusion in this study, if they presented with heart failure symptoms at NYHA stage I, II, or III with either reduced systolic left ventricular ejection fraction (HFrEF) or preserved systolic left ventricular ejection fraction (HFpEF) due to (1) ischemic cardiomyopathy (ICM, *n* = 44, coronary artery disease invasively diagnosed by coronary angiography with or without history of myocardial infarction); (2) dilated cardiomyopathy (DCM, *n* = 39, left or biventricular enlargement, exclusion of coronary artery disease, or arterial hypertension); or (3) hypertensive heart disease (HHD, *n* = 31, left ventricular hypertrophy in response to uncontrolled arterial hypertension, exclusion of coronary artery disease). Patients who underwent acute decompensations, an acute coronary syndrome, or a change in NYHA class within the last 3 month were excluded from the study as well as patients with acute infections, active neoplastic diseases, relevant anemia, or terminal renal failure. Detailed patients' characteristics including NYHA functional class, cardiovascular risk factors, medication, or selected laboratory parameters are given in Table 1. Laboratory parameters as well as electrocardiograms were measured according to internal and international standard procedures. Echocardiography was performed using a Philips ultrasound system (iE33, Philips, Germany) by experienced cardiologists not involved in the study. Blood samples were centrifuged at 2383 g for 25 min immediately after collection. Supernatants were stored at −80°C until further analysis and strictly prevented from repeated freeze-thaw cycles to minimize degradation of proteins. All measurements were performed in duplicate. For statistical analysis, mean values of the ELISA results of each patient were used.

### 3.3. Statistics

All data are expressed as mean ± standard deviation (SD). All statistical analyses were performed with IBM SPSS statistics, version 22.0 (IBM Inc.). Kruskal-Wallis test was used to test for significant differences in the results of quantitative ED-A^+^ Fn measurement between different groups. Bivariate correlations between parametric variables were assessed by Pearson's correlation coefficient. A *p* value ≤ 0.05 was defined to be statistically significant. To test the value of ED-A^+^ Fn as a predictor of ischemic heart failure etiology, multivariate analysis (stepwise multiple regression) was performed using a binary logistic model (backward elimination method: Wald). Heart failure etiology (ischemic versus nonischemic) was defined as dependent variable. Age, NYHA class, LVEF, BNP, and ED-A^+^ Fn were included into the model in the first step. Then, multistep backward elimination (removal threshold *p* > 0.10) of independent variables was carried out. *p* values ≤ 0.05 were considered to be statistically significant.

## 4. Results

### 4.1. ELISA Procedure

**Figure 1:**
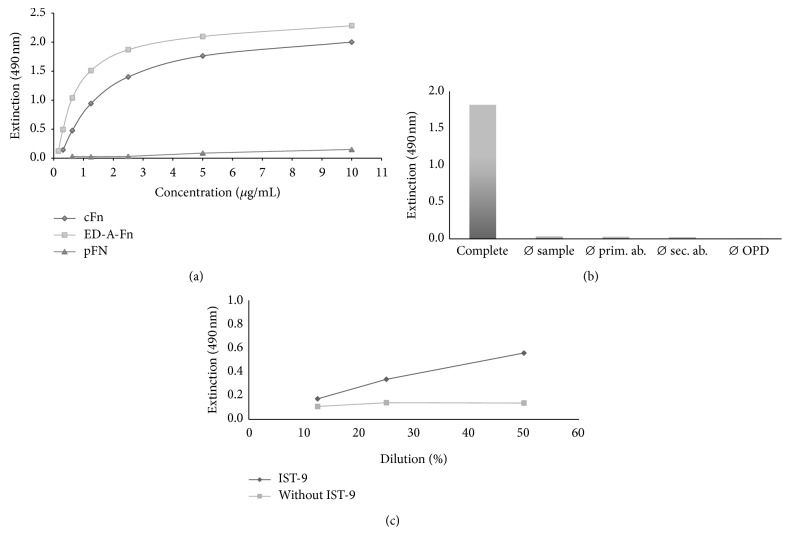
Establishment of the specific ELISA for quantification of ED-A^+^ Fn: Evaluation of different ED-A^+^ Fns as molecule for standard quantification (a) and exclusion of cross-reactions to pFn (a), between different components of the ELISA setup (b), and to endogenous immunoglobulins (c).

Generation of a standard curve revealed that pure ED-A^+^ Fn displays a slightly higher extinction as compared to cFn at the same concentration (Figure 1(a)). Both proteins revealed a linear range of concentration, which can be used for quantification of unknown samples. Because of the excellent characterization of the purified ED-A^+^ Fn, we used this Fn preparation as standard protein in the following experiments.

To guarantee the specific detection and quantification of exclusively ED-A^+^ Fn, binding of plasma fibronectin has to be excluded. This was tested by measuring pFn in a range of concentration equal to the range of concentration of ED-A^+^ Fn and cFn for the standard curve. As illustrated in Figure 1(a), exclusively cFn, either from melanoma cells or from fibroblasts, and not pFn is measured by using the newly established ELISA, which is therefore appropriate for specific quantification of ED-A^+^ Fn.

In order to evaluate the specificity of the established ED-A^+^ Fn ELISA, it is mandatory to exclude cross-reactions between the components of the test system. For this, a “complete” protocol was compared with 4 modified experimental setups as described above. As shown in Figure 1(b), we could observe that the development of a distinct absorbance is only visible when the complete ELISA is carried out excluding false positive results due to unspecific binding of a single component of the assay.

To exclude cross-reactivity of the donkey-*α*-mouse secondary antibody with endogenous human immunoglobulines, human test serum samples were used in a complete ELISA setting compared to an assay protocol, in which the primary antibody IST-9 was omitted. As a result, no reactivity is seen independent of serum concentration when the detection antibody was absent. This speaks well for a highly specific reactivity of the secondary antibody exclusively to the primary antibody IST-9 (mouse immunoglobulin) (Figure 1(c)).

### 4.2. ED-A^+^ Fibronectin as Novel Serum Biomarker for Cardiac Tissue Remodeling

**Figure 2:**
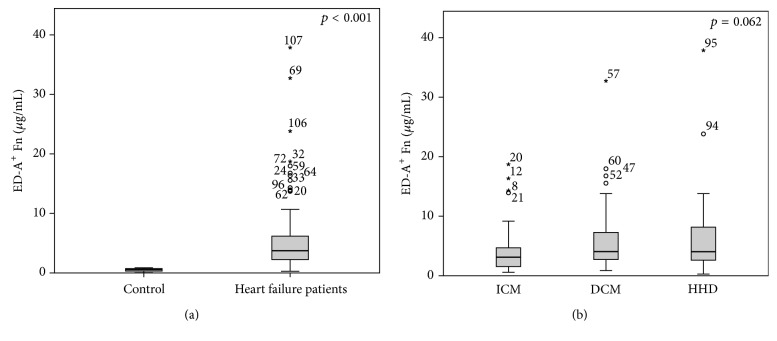
Quantification of ED-A^+^ Fn in a cohort of heart failure patients: ED-A^+^ Fn is significantly (*p* < 0.001) increased in serum of heart failure patients compared to healthy controls (a) but no correlation between ED-A^+^ Fn and different heart failure etiologies was observable (*p* = 0.062) (ICM: ischemic cardiomyopathy; DCM: dilated cardiomyopathy; HHD: hypertensive heart disease).

**Figure 3:**
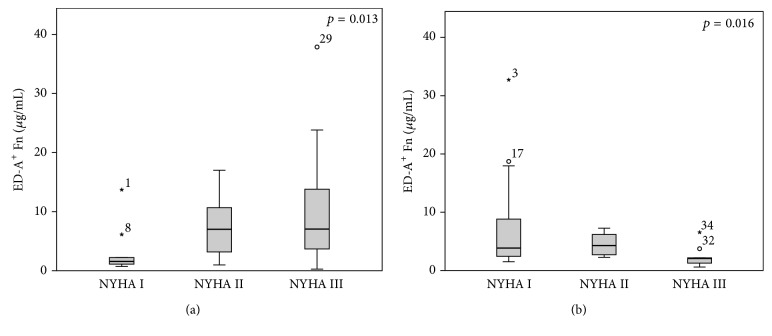
Correlation between ED-A^+^ Fn and NYHA functional class: significantly (*p* = 0.013) increasing levels of ED-A^+^ Fn with higher NYHA class in ischemic cardiomyopathy (a) and significantly (*p* = 0.016) decreasing levels in dilated cardiomyopathy (b).

To preliminarily evaluate if ED-A^+^ Fn might be a promising novel serum biomarker of pathologic cardiac tissue remodeling, we determined serum concentrations in patients suffering from heart failure (*n* = 114) due to different etiologies in comparison to serum samples from 12 healthy volunteers (mean age: 35 years; 75% male and 25% female) without any known cardiovascular pathology. Baseline characteristics of heart failure patients are given in Table 1. The totality of patients showed significantly increased serum levels of ED-A^+^ Fn (5.58 ± 5.97 *μ*g/mL) compared to healthy volunteers (0.52 ± 0.26 *μ*g/mL) (*p* < 0.001; Figure 2(a)). When comparing the different groups of etiology within the heart failure patients, no significant differences in the ED-A^+^ Fn serum concentrations could be observed (*p* = 0.062; Figure 2(b)). There were significantly lower ED-A^+^ Fn concentrations in the ICM group (4.27 ± 4.12), compared to the DCM group (6.24 ± 6.11; *p* = 0.027). There were no differences in the serum levels of ED-A^+^ Fn in relation to the NYHA functional classes I to III when analyzing the complete heart failure population collectively (*p* = 0.618). After dividing patients according to their etiology group, an increase in ED-A^+^ Fn serum levels with rising NYHA class could be demonstrated for patients with ICM (*p* = 0.013; Figure 3(a)). In contrast, patients with DCM showed converse findings (*p* = 0.016; Figure 3(b)). For the HHD group, no differences in the ED-A^+^ Fn serum levels in association with NYHA class occurred (*p* = 0.207).

**Figure 4:**
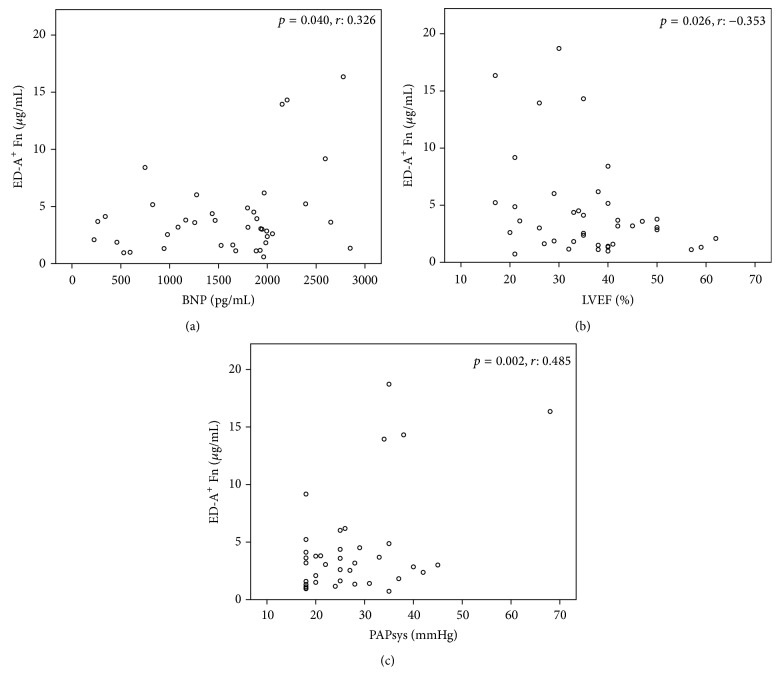
Serum levels of ED-A^+^ Fn in ICM: Significant positive correlation between ED-A^+^ Fn and BNP (*p* = 0.040, *r* = 0.326) (a), significant negative correlation between ED-A^+^ Fn and LVEF (*p* = 0.026, *r* = −0.353) (b), and significant positive correlation between ED-A^+^ Fn and PAPsys (*p* = 0.002, *r* = 0.485) (c).

For a subset of heart failure patients, for whom the serum brain natriuretic peptide (BNP) levels were available (*n* = 100), we performed a Pearson correlation analysis. Here, we could not show any correlation between serum BNP and serum ED-A^+^ Fn levels for the whole patient group (*p* = 0.42, *r*: 0.081). Only for the subgroup of heart failure patients suffering from ICM (BNP available in 40 out of 44 patients), there was a significant positive correlation between BNP and ED-A^+^ Fn serum levels (*p* = 0.040, *r*: 0.326; Figure 4(a)). For the other groups (DCM and HHD), there were no significant correlations.

We further divided the heart failure patients in a group exhibiting sinus rhythm, a group showing atrial fibrillation, and a group with pacemaker rhythm according to the performed electrocardiograms at rest. We could not find any significant differences in the determined ED-A^+^ Fn serum levels between these groups (*p* = 0.775).

To analyze associations between ED-A^+^ Fn serum concentration and left ventricular remodeling and function, we focused on the following echocardiographic surrogate parameters: left ventricular ejection fraction (LVEF in %), left ventricular end-diastolic diameter (LVEDD in mm), left atrial diameter (LAD in mm), and pulmonary artery pressure in systole (PAPsys).

With respect to LVEF, there were no significant differences between patients showing an LVEF ≤ 30% (7.07 ± 7.04 *μ*g/mL) compared to those with an LVEF > 30% (5.16 ± 5.7 *μ*g/mL) (*p* = 0.182). When analyzing the total cohort of heart failure patients, we could not reveal any significant correlation between LVEF and serum ED-A^+^ Fn. Between the three subgroups according to disease etiology, there were no relevant differences in LVEF (mean LVEF in the ICM group = 36 ± 11.24%; mean LVEF in the DCM group = 40 ± 12.83%; and mean LVEF for the HHD group = 40 ± 12%). In the ICM subgroup of patients, there was a significant negative correlation between LVEF and ED-A^+^ Fn serum levels (*p* = 0.026, *r*: −0.353; Figure 4(b)). For LVEDD, we could not observe any association with the serum levels of ED-A^+^ Fn, neither when comparing patients with normal and dilated left ventricles (LVEDD > 56 mm), nor when performing correlation analysis for the whole cohort or different subgroups, respectively. The median LAD parameter in the heart failure patients investigated in this study was 46 mm. In patients that exhibited an LAD > 46 mm, in trend elevated ED-A^+^ Fn concentrations could be determined (*p* = 0.051). Within the totality of patients, there was no correlation between LAD and serum ED-A^+^ Fn. But, in contrast to all other subgroups, within the ICM group, a positive correlation was observable (*p* = 0.008, *r*: 0.431). Patients with normal PAPsys did not show any differences in serum ED-A^+^ Fn compared to those with elevated PAPsys when analyzing the total patient cohort (*p* = 0.426). When looking into the etiological subgroups, we could evidence a strong positive correlation between PAPsys and serum ED-A^+^ Fn in ICM patients (*p* = 0.002, *r*: 0.485; Figure 4(c)).

**Figure 5:**
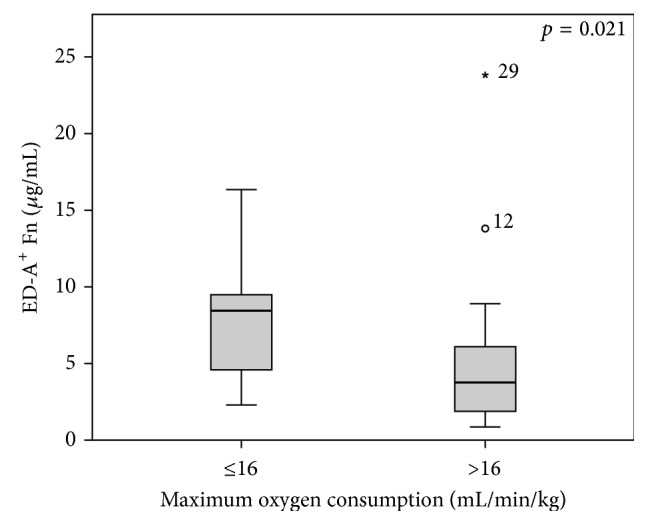
Levels of ED-A^+^ Fn in context to spiroergometric assessment: Significantly (*p* = 0.021) increased levels of ED-A^+^ Fn in heart failure patients with maximum oxygen consumption less than 16 mL/min/kg.

Finally, we investigated possible associations between serum ED-A^+^ Fn and spiroergometric data. Within the totality of heart failure patients, we could show that there are decreased ED-A^+^ Fn levels in patients with a maximum oxygen consumption of more than 16 mL/min/kg compared to those who achieved values of ≤ 16 mL/min/kg (*p* = 0.021; Figure 5). For maximum achieved wattage, no significant associations could be observed (data not shown).

### 4.3. Multivariate Analysis

Against the background of the interesting correlations between ED-A^+^ Fn serum levels and clinical parameters especially in the subgroup of heart failure patients suffering from ICM, we tested the value of serum ED-A^+^ Fn as a predictor of ischemic etiology of heart failure by performing a multivariate analysis. Age, NYHA class, LVEF, BNP, and ED-A^+^ Fn were entered into the analysis as independent variables. After backward elimination, only age (Wald: 8.507, OR: 0.937, 95% CI: 0.896–0.979, and *p* = 0.004) and ED-A^+^ Fn (Wald: 4.209, OR: 1.125, 95% CI: 1.005–1.258, and *p* = 0.040) but not NYHA class, LVEF, and serum BNP levels could be evidenced to be independent predictors of an ischemic heart failure etiology. Thus, the probability of an ischemic etiology decreases with rising subject age and increases with rising ED-A^+^ Fn serum levels.

## 5. Discussion

Fibronectin in general is an abundant soluble constituent of human serum and also other body fluids. Serum mostly contains soluble plasma fibronectin (pFn) whereas less-soluble cellular fibronectin (cFn) is virtually absent. In contrast to pFn, cFn contains the alternative spliced ED-A, ED-B, and IIICS domains [38–40]. To determine the diagnostic value of ED-A^+^ Fn in cardiac diseases, we initially established a novel ELISA method for quantification of soluble ED-A^+^ Fn in serum samples. Already in the early 1990, first ELISA for ED-A domain containing cFn were developed using, for instance, the antibody DH1 and it could be demonstrated that this Fn isoform in serum/plasma may have a special importance for diagnosis and prognosis in different malignant and benign pathologies [41–43]. The protocol for quantification of soluble ED-A^+^ Fn applied in this study entails a gelatin based ELISA as already described for quantification of pFn by Selmer and coworkers in 1984 [35]. We were able to optimize the method regarding a specific detection of solely ED-A^+^ cFn by using an antibody specific to the ED-A domain of Fn for detection [40]. Additionally, we could prove the specificity of the newly developed ELISA protocol by comparing the extinction of pFn with that of ED-A^+^ Fn at the same range of concentration, whereby pFn was not detectable. Differences in the extinction of cFn isolated from human foreskin fibroblasts with an unknown procedure of purification (Sigma-Aldrich, Missouri, USA) and ED-A^+^ Fn from melanoma cell line supernatant (Sirius Biotech, Genoa, Italy) may be due to different methods of purification. Both proteins could be used as standard proteins because the standard curves exhibit a linear range for quantification. Nevertheless, we decided to use the latter because this protein was purified by using an IST-9 Sepharose column. Cross-reactivity between the secondary antibody and endogenous immunoglobulines was excluded by using a highly absorbed donkey-*α*-mouse antibody. After performing several experiments for optimization of the ELISA setup, the lowest measurable concentration of ED-A^+^ Fn was 0.078 *μ*g/mL.

Already in 1995, Ylatupa and his group asked if ED-A^+^ Fn is a potential new biomarker for diseases with extracellular remodeling. They were able to describe a significant elevation of ED-A^+^ Fn in plasma of patients with malignant tumors [42]. In the following years, increased release of ED-A^+^ Fn to serum/plasma was shown to be associated with rheumatoid vasculitis/arthritis, diabetes, preeclampsia, urinary bladder cancer, renal cell carcinoma, gastrointestinal and head and neck cancer, and parenchymal hematoma after thrombolytic therapy in acute ischemic stroke [44–52]. In the current study, our group was able to demonstrate that ED-A^+^ Fn is valuable biomarker also for cardiovascular (extracellular matrix) remodeling by proving that heart failure patients exhibit significantly increased serum levels compared to healthy controls. In correspondence to that, van Keulen and coworkers could show that ED-A^+^ Fn may act as biomarker for stability of atherosclerotic plaques [53]. In addition, there is also growing evidence on the functional role of the ED-A domain of fibronectin in the process of myocardial remodeling by mediating the transdifferentiation of fibroblast to MyoFb as well as by activating vascular smooth muscle cells in the tunica media of vessel structures contributing to vascular remodeling, for example, in allograft vasculopathy or classic atherosclerosis [14, 54, 55]. Furthermore, ED-A^+^ Fn is able to activate the toll-like receptor 4 (TLR4) in macrophages, dendritic cells, and fibroblasts with subsequent NF-kB activation, MMP expression, and release of proinflammatory cytokines [56–58].

Although there were no relevant differences between the different etiology groups investigated in the current study, especially in the subgroup of heart failure patients suffering from ICM, interesting associations between the state of disease and the level of soluble serum ED-A^+^ Fn could be demonstrated, for example, a positive correlation with NYHA functional class, serum BNP levels, left ventricular dysfunction, or systolic pulmonary artery pressure. Taken together, increased serum ED-A^+^ Fn was associated with a more advanced case. The association between elevated systolic pulmonary artery pressure and ED-A^+^ Fn serum levels as observed in the current study might arise from vascular remodeling in the lung. Thus, the finding might be interpreted as a phenomenon not directly but secondarily linked to heart failure in the sense of pulmonary hypertension due to left heart disease. To our best knowledge, there is no further study available in the literature investigating serum ED-A^+^ Fn in heart failure. The potential impact of ED-A^+^ Fn as a diagnostic or even prognostic biomarker in heart failure patients has to be reevaluated in further studies including larger patient numbers. Nevertheless, the fact that ED-A^+^ Fn is an independent predictor of an ischemic heart failure etiology as proven by multivariate analysis in this study strongly supports a certain role of the molecule especially in the process of postischemic left ventricular remodeling. This finding should encourage performing additional studies including in vitro as well as animal experiments addressing the pathobiological function of ED-A^+^ Fn in cardiac hypoxia and ischemia.

In contrast to ED-A^+^ Fn, there are a variety of studies focusing on fetal Tn-C variants in the circulation of patients with different cardiovascular diseases like hypertension, myocarditis, or different cardiomyopathies, respectively [13, 17, 23, 59]. For all three heart failure etiologies investigated in the current study, namely, ICM, DCM, and HHD, increased serum levels of B and/or C domain containing Tn-C with associations with disease severity in the broadest sense could be proven by several groups [13, 16, 17, 19, 23, 60, 61]. In contrast, for ED-A^+^ Fn we could show significant correlations only in the ICM group. The question arises on why ICM plays a certain role with respect to ED-A^+^ Fn liberation into the circulation compared to other cardiomyopathies like DCM or HHD. One possible explanation is the nature of fibrosis as an important component of pathologic cardiac tissue remodeling: in DCM or HHD, reactive fibrosis can be detected, whereas in ICM replacement or reparative fibrosis is present, generally in response to a myocardial infarction which leads to a fibrotic scar [2, 4]. Within this process, the activation of fibroblasts as well as the transdifferentiation to myofibroblasts is of great functional importance. ED-A^+^ Fn has been proven to be an essential cofactor during myofibroblast development [14, 54]. This might be a possible explanation for the particular finding that ED-A^+^ Fn seems to have a special role in heart failure patients due to ICM. To elucidate these open questions in detail on the functional level must be the object of further studies. Anyhow, a very interesting finding of the current study is that there seem to be differences between the disease-associated tissue deposition of ED-A^+^ Fn and its liberation into the circulation. Our group could recently show that the molecule is deposited in diseased cardiac tissue in spatial association with vascular structures and with interstitial fibrosis in a variety of diseases, for example, DCM, ICM, and valvular heart disease, or in an animal model of cardiac rejection, respectively [26–32]. Surprisingly, only in ICM an association with disease severity could be shown for serum ED-A^+^ Fn in this study. Based on these considerations, one can assume that tissue deposition of ED-A^+^ Fn and its liberation into the circulation differ and may be independently regulated processes. Although it is demonstrated that there is a disease related increased release of cFn into serum/plasma, the mechanisms leading to enhanced concentrations are not fully understood up to now and may differ between pathologies. In general, also cFn is synthesized as a soluble molecule gaining insolubility during fibrillogenesis as an active cellular process involving the *α*5*β*1 integrin receptor. Furthermore, Fn matrix assembly is a continuous process with a steady state between Fn polymerization and turnover (for review see [62]). Therefore, all factors influencing the steady state with reduced matrix assembly may have also impact on serum concentration: increased synthesis, changes in integrin expression, altered endocytosis, and proteolysis/matrix degradation during tissue remodeling must be taken into account. Hitherto, these questions cannot be answered and should be further addressed by comparing tissue and serum ED-A^+^ Fn levels intraindividually for the different disease groups in continuative studies.

As already mentioned above, not in serum but in diseased tissue, a distinct reexpression of ED-A^+^ Fn could be demonstrated in several neoplastic, inflammatory, or cardiovascular disorders qualifying the molecule as an excellent target structure for an antibody based delivery of bioactive payloads or diagnostic agents using the human antibody F8 [15, 32, 63, 64]. In this context one could imagine that the individual level of soluble in particular serum ED-A^+^ Fn might be of importance since the systemically administered antibody based agent could possibly bind to circulating molecules hindering the antibody to accumulate in the target tissue, where ED-A^+^ Fn is abundantly expressed and deposited. Thus, a pretherapeutic measurement of soluble ED-A^+^ Fn could become an important component of individualized therapy planning.

## Additional Points

*Highlights*. (i) In summary, the current study could clearly show that ED-A^+^ Fn is a promising biomarker in cardiovascular diseases, especially in heart failure patients suffering from left ventricular dysfunction due to an ICM. In multivariate analysis, ED-A^+^ Fn could be identified as an independent predictor of an ischemic heart failure etiology. (ii) We presented a valid ELISA method for serum detection of the molecule, which could be applied for further studies investigating the diagnostic and prognosis-predicting value of ED-A^+^ Fn in different disorders. (iii) Furthermore, in case of targeted delivery of immunocytokines or antibody-drug/contrast-medium-conjugates directly into the diseased tissue using human antibodies specific to ED-A^+^ Fn, the pretherapeutic determination of circulating ED-A^+^ Fn levels could help to increase the extent of tissue accumulation and thereby the efficiency of treatment or imaging. (iv) From the functional point of view, it will be of great interest to elucidate the underlying mechanisms and the potential biological importance of ED-A^+^ Fn liberation from diseased tissue into the circulation in different, not only cardiovascular, diseases.* Limitations of the Study*. There are some important limitations of the current study that should be clearly mentioned: (i) The 12 healthy subjects, which served as control group, were not optimal since they were significantly younger compared to the heart failure patients. Due to the fact that serum ED-A^+^ Fn concentrations might be altered in association with an increasing subject age, in further studies age-matched controls should be used. (ii) The number of heart failure patients in this retrospective study is limited, especially when looking into the different etiological subgroups. Thus, the potential impact of ED-A^+^ Fn as a diagnostic or even prognostic biomarker has to be reevaluated in further prospective studies including larger patient numbers.

## Figures and Tables

**Table 1:** Detailed patients' characteristics.

Patients' characteristics			*n*
Age (years)		61 ± 13	114

NYHA class: I/II/III in % (*n*)		33% (*n* = 37)/37% (*n* = 41)/30% (*n* = 34)	112

Heart failure genesis	Ischemic, % (*n*)	39% (*n* = 44)	114
	Dilated, % (*n*)	34% (*n* = 39)	
	Hypertensive, % (*n*)	27% (*n* = 31)	

Body weight (kg)		91 ± 18	106

Systolic blood pressure (mmHg)		137 ± 22	110

Diastolic blood pressure (mmHg)		86 ± 14	110

Comorbidities, % (*n*)	Hypertension	82% (*n* = 93)	113
	Hyperlipidemia	55% (*n* = 63)	114
	Diabetes mellitus	29% (*n* = 33)	113

Comedication, % (*n*)	Statin	57% (*n* = 63)	111
	ACE-inhibitor	78% (*n* = 87)	111
	Beta-blocker	97% (*n* = 109)	112

Laboratory values, mean ± SD	Creatinine (*μ*mol/L)	118 ± 76	112
	Cholesterol (mmol/)	5 ± 1.4	102
	LDL-cholesterol (mmol/L)	3 ± 1.2	102
	BNP (pg/mL)	1591 ± 796	100
	Hemoglobin (g/dL)	14 ± 1.9	114
